# The Role of Project Management Office in Public Health: A New Approach for Establishment in Iran

**Published:** 2017-03

**Authors:** Morteza BAGHERPOUR, Asma ERJAEE

**Affiliations:** 1. Dept. of Industrial Engineering, Iran University of Science and Technology, Tehran, Iran; 2. Dept. of Pediatrics, Shiraz University of Medical Sciences, Shiraz, Iran

## Dear Editor-in-Chief

Healthcare management and public health have been significantly grown over decades. Based on report presented by Center for Medicare and Medicaid Services (CMS), healthcare spending will grow to about 19.8% of US GDP by 2020. Other countries have spent huge investment on health sector as well. Therefore, in order to improve public health services and with the aim of attaining better performance relevant to public health, several healthcare projects must be newly defined.

Key factor for success here is how to manage the projects undertaken through a well-organized monitoring system. The term “project” is referred to an endeavor which has some attributes such as clear scope of work, uniqueness of services for each project, temporarily efforts in a specific period of time (planned start and finish are known). Therefore, the need for development of project management systems in public health can be truly seen. Examples of some projects can be presented as: public vaccinations/immunization programs, environmental health, establishment of telemedicine or family medicine, public policies for health programs, defining an integrated medical record system for public sectors or any intervention program for prevention of a serious disease and any other projects defined in order for improving current status of hospitals and medical centers.

For instance, health dental program in school (1) and research management information system (2) previously suggested as public health programs that each considered as an individual project. On the other hand, project management systems have clearly intended to manage scope, time, cost, quality and risk for the projects undertaken. In order for managing all projects simultaneously, in industrial sectors such as construction projects, a Project Management Office (PMO) has been established for managing all the projects and assure project’s success rate as well. Several PMOs nowadays can be found in many heavy intensive industries (3). Only 19% of projects delivered on time, 18% delivered within budget and 15% produced high-quality deliverables (4). This confirms our suggestion for running a PMO.

In spite of growth in PMO establishments through industrial sectors, however, the PMO in health and public sector has not been increasingly employed. Just a few instances of application of PMO in U.S. hospitals can be found. Theoretically, it has been suggested to use project management in health research projects in Iran (5). The need for establishment of a well-organized was summarized PMO through public health is crucial, from both theoretical and practical point of views. This PMO should perform the following functions as given below:

- Definition of the essential projects and prioritization of the projects based on level of importance and their contributions in public health programs
- Utilization of well-organized procedures and protocols for initiating, planning, controlling and monitoring of whole the projects carried out.
- Monitor all projects according to time, cost, risk and quality performance indicators
- Knowledge inspiration and management through development of the project lessons learned
- Performance evaluation and assessment of sub-sections related to public health programs such as hospitals and clinical units.
- Draw future trends of public health and analyze success rate of the projects carried out periodically.
- Make managerial and strategic decisions for managing time, cost and quality
- Establish key performance indicators for performance evaluation and analysis

This type of PMO can be established in two ways. The first approach is to define a supportive PMO, which its function can be limited to providing project management supports and services. By this logic, all clinical units and hospitals have to be established a supportive PMO even partially. The latter should forward all information to an integrated supportive PMO to manage all information and generate further integrated reports. In Table 1, a timetable for PMO establishment is given within two years.

**Table 1:** Timetable for PMO establishment

**PMO establishment**	**6 months**	**6 months**	**6 months**	**6 months**
PMO design	X			
Training programs	X	X		
PMO implementations		X	X	
PMO integration			X	X
Dashboard development				X

Once the supportive PMO becomes established now it has time to organize a managerial PMO which obtained enough maturity levels for making complicated managerial decisions (the second phase). By this approach, all top-level managers have to contribute through PMO. Finally, a well-organized public health dashboard and alarming system would be an advantage behind implementation of this system. Establishment of PMO in public health we are moving toward smart health system as the next stage. This system will satisfy and make a balance among all the needs and expectations of public health’s stakeholders.
